# Non- medical prescribing in Australasia and the UK: the case of podiatry

**DOI:** 10.1186/1757-1146-3-1

**Published:** 2010-01-05

**Authors:** Alan M Borthwick, Anthony J Short, Susan A Nancarrow, Rosalie Boyce

**Affiliations:** 1 School of Health Sciences, University of Southampton, Highfield, Southampton, UK; 2 School of Public Health, Queensland University of Technology, Brisbane, Australia; 3 Centre for Health and Social Care Research, Faculty of Health and Wellbeing, Sheffield Hallam University, UK; 4 School of Pharmacy, University of Queensland, Brisbane, Australia

## Abstract

**Background:**

The last decade has witnessed a rapid transformation in the role boundaries of the allied health professions, enabled through the creation of new roles and the expansion of existing, traditional roles. A strategy of health care 'modernisation' has encompassed calls for the redrawing of professional boundaries and identities, linked with demands for greater workforce flexibility. Several tasks and roles previously within the exclusive domain of medicine have been delegated to, or assumed by, allied health professionals, as the workforce is reshaped to meet the challenges posed by changing demographic, social and political contexts. The prescribing of medicines by non-medically qualified healthcare professionals, and in particular the podiatry profession, reflects these changes.

**Methods:**

Using a range of key primary documentary sources derived from published material in the public domain and unpublished material in private possession, this paper traces the development of contemporary UK and Australasian podiatric prescribing, access, supply and administration of medicines. Documentary sources include material from legislative, health policy, regulatory and professional bodies (including both State and Federal sources in Australia).

**Results:**

Tracing a chronological, comparative, socio-historical account of the emergence and development of 'prescribing' in podiatry in both Australasia and the UK enables an analysis of the impact of health policy reforms on the use of, and access to, medicines by podiatrists. The advent of neo-liberal healthcare policies, coupled with demands for workforce flexibility and role transfer within a climate of demographic, economic and social change has enabled allied health professionals to undertake an expanding number of tasks involving the sale, supply, administration and prescription of medicines.

**Conclusion:**

As a challenge to medical dominance, these changes, although driven by wider healthcare policy, have met with resistance. As anticipated in the theory of medical dominance, inter-professional jurisdictional disputes centred on the right to access, administer, supply and prescribe medicines act as obstacles to workforce change. Nevertheless, the broader policy agenda continues to ensure workforce redesign in which podiatry has assumed wider roles and responsibilities in prescribing.

## Introduction

Recent health policy reforms, underpinned by the 'ongoing influence of a neo-liberal and managerialist agenda', have clearly enabled an extension in the role boundaries of the allied health professions (AHPs) in both the UK and Australasia [1,2]. In the past, paramedical advances in prescribing of medicines had been largely constrained through the exercise of medical power, often described as professional autonomy [3-5]. Professional autonomy has been widely explored in relation to the medical and allied health professions [6,7]. In broad terms, professional autonomy is taken to represent the 'legitimated control that an occupation exercises over the organisation and terms of its work'[6].

Medicine has often been considered as an exemplar of the autonomous profession, characterised by its authority, and hegemony over the other health professions [2,4,6,8-11]. Several explanations have been provided to indicate the way in which medical professionalism was gradually extended from self-regulatory authority to control over the knowledge base, role boundaries and status of other healthcare professions [7,12-18]. The concept of medical dominance was instrumental in capturing the establishment of hegemonic authority by medicine within a hierarchical arrangement of power in healthcare [4,7,10,14,16,19]. One aspect of this theory has been subject to criticism, however. It appears to accept as inescapable the submission of other healthcare professions to medical hegemony, without acknowledging their own aspirations [14,16,17]. Indeed, evidence continues to accumulate which suggests a fundamental shift in medical dominance [1,11,20-24]. Larkin [17] examined the case for a significant transition in inter-professional relations towards co-equal partnerships, in a shifting profession-state arena. Now, government reforms are thought to 'encourage' a 'new type of professionalism - that is not based upon exclusion, control and special status'. Nevertheless, evidence for any significant displacement of medical dominance remains elusive in the absence of any identifiable alternative, in spite of the rhetoric of modernisation [17].

Healthcare policy and reorganisation in both Australasia and the UK has been taken to indicate a decline in medical dominance, particularly over the last two to three decades [1,2,17,20,25,26]. One central feature of the dominance of medicine has been the near exclusive right to prescribe medicines. In both Australasia and the UK legislation has, for many years, recognised medicine (and dentistry) as sole recipients of the right to access, administer, supply and prescribe from a full range of available medicines, justified on the basis of the special knowledge, education and skill of the profession. Other healthcare professions, such as nursing and the allied health professions, initially excluded, have gradually been able to secure increasing rights to access, supply, administer and finally prescribe medicines, against a backdrop of medical resistance [27]. By tracing the emergence and development of allied health 'prescribing', from early rights to administer and supply certain restricted category medicines, to the more recent forms of actual prescribing, it is possible to map the changing relationships between the profession of medicine, government and the allied health professions.

In this paper, podiatry will serve as a case exemplar. In light of its long engagement with medicine and government Health Departments over the right to use restricted category medicines, it enables a socio-historical account to be constructed which adequately illustrates the gradual impact on the profession of changing health policy reform as a response to wider challenges to the provision of healthcare in both Australasia and the UK.

## Methods

This data in this paper were constructed from a range of documentary data sources, primarily derived from published sources in the public domain, supported by access to unpublished material in private possession. Policy documents, consultation papers, minutes of meetings, press releases, publications from professional and regulatory bodies and legislative sources were included. Documents were sourced from within Australasia and the UK, including both State and Federal sources in Australia. These data were used to construct a socio-historical account of the development of contemporary UK and Australasian podiatric access, supply, administration and prescription of medicines, grounded in a health policy context. Theory derived from the sociology of the professions underpinned the socio-historical analysis.

## Results

### AHP prescribing in the UK and Australasia

Whilst formal prescribing by non-medically qualified allied health professionals is a new and fairly limited phenomenon in both the UK and Australasia, several of the smaller professions, most notably podiatrists and optometrists, have been accustomed to exercising legal rights to access, supply, administer and sell a restricted range of medicines over several decades [28-38]. In other cases, such as physiotherapy and radiography, legal rights to the supply or administration of restricted category medicines have either been available only since 2005 (in the UK), or, in the case of Australian physiotherapy, not yet recognised [39,40]. Where rights do exist, the mode of use is often different, ranging from specialist practitioners working in fields such as musculo-skeletal care (in the case of physiotherapy) to more general use, as in the case of podiatry [28,41].

From a socio-historical viewpoint, since the 1960s, the emergence of supply and administration rights to certain restricted medicines, in the wake of changing legislation, might well be viewed as part of the broader 'professional project' of podiatrists, optometrists and others, and central to their desire to confirm professional autonomy and independence in practice [33,42]. Podiatry and optometry have, in the past, been distinguished from other allied health professions on the grounds of their independence from hospital practice, and thus relative freedom from immediate supervision by the medical profession [14].

Radiography and physiotherapy, conversely, emerged more clearly as hospital employees working in direct support of the medical profession, on a daily basis [14]. It is also clear that not all allied health professions are likely to become active prescribers, or aim to secure legal exemptions allowing limited access to medicines, possibly such as arts therapists.

In order to adequately understand the effect of managerialist health policy reforms on the transition in podiatric role boundaries, it is necessary to trace the chronological context of change. As a result, the context of podiatric prescribing must take into account the broader privileges associated with the legal right to access, administer, sell and supply specified medicines that are otherwise restricted (that is, those that fall within in the 'prescription only' and 'pharmacy only' categories of medicine in the UK and New Zealand, and those within the Schedule 3,4 and 8 categories in Australia), as well as the actual 'prescribing' mechanisms involved in 'supplementary' and 'independent' versions [43], or the recent freedom granted in Victoria (Australia) to podiatrists under the Health Professions Registration Act of 2005.

In the UK, prior to the advent of supplementary prescribing or patient group directions podiatrists had 'accessed', 'supplied/sold' and 'administered' restricted category medicines through authority granted by statutory instruments. A statutory instrument is a form of 'delegated' legislation, allowing exemptions to be made to the provisions of established 'primary' legislation (in this case the Medicines Act of 1968), without the need to repeal the entire Act [44]. Similarly, in Australia amendments to State or Territory legislation, such as the Health (Drugs & Poisons) Regulation 1996 (Queensland), grants the profession specific rights under the authorisation of the Health Minister.

### Medicines and the allied health professions: a socio-historical context

#### 1. The UK

In the early 1960s, public confidence in healthcare was undermined by the unforeseen complications arising from the use of the approved medicine thalidomide (causing teratogenic deformities in the offspring of women pregnant during the drug treatment) and resulted in a major review of the adequacy of existing medicines legislation [45,46]. New, unifying, legislation designed to supercede the existing provisions was put in place, covering several features, such as the manufacture, and marketing or licensing of medicines, alongside new mechanisms for regulating access, administration, sale and supply [28,46].

A Medicines Commission was also established, with a remit which included a role in determining whether submissions for exemptions by professional groups excluded under the new legislation (The Medicines Act 1968) would be accepted or rejected [28]. Under the new system, medicines were classified as 'prescription only', 'pharmacy only' or 'general sales list'. 'Prescription only' medicines were available only to 'appropriate practitioners', who were identified as doctors of medicine, dentists and veterinary practitioners (Part III of the 1968 Act). However, podiatrists had already been using several medicines caught up in the re-classification, and found they no longer had rights to their use. As a result, the profession was forced to utilise the new arrangements, and seek legally recognised exemptions to gain access to medicines already part of normative practice - arrangements which proved difficult to navigate without opposition.

Within a decade, the political landscape began to change dramatically, as policy reforms throughout the 1980s gradually made possible greater access to restricted medicines. By the mid-1980s the Thatcher Government, a neo-liberal, market oriented conservative administration, introduced a series of reforms which impacted directly on the autonomy of the medical profession and its exclusivity in the delivery of certain services, including the supply of medicines [47,48]. First, 'indicative' prescribing was introduced, limiting doctors prescribing habits in the interests of fiscal probity, whilst acting to diminish medical power [49]. Within a short time, plans to grant specialist nurses and midwives enhanced 'prescribing' rights were unveiled [50,51].

Nevertheless, these changes did not mean progress towards AHP prescribing would be unproblematic. On the contrary, role boundary disputes arose, creating obstacles to change [28,50-52]. Yet, by 1999 a new policy direction was announced in the 'Crown Report' review of non-medical prescribing, which was rapidly accepted by Government [43]. Pressure for change stemmed from a number of converging dilemmas facing the future of health care provision; an ageing population, changing disease profiles and a reduced workforce, coupled with a looming crisis in healthcare recruitment and retention, fiscal constraint and the challenge of European Union Working Time Directives. A new policy agenda emphasising new ways of working, role substitution and workforce redesign emerged [53-58]. Key nurse and allied health professional groups would, it was envisioned, emerge as genuine 'prescribers' [43]. Two new categories of prescriber were defined - 'independent' and 'dependent' (later 'supplementary'), reflecting a new level of autonomy for selected allied health professionals. Independent prescriber status was suggested as possible for five named professional groups, including extended scope physiotherapists, optometrists and podiatrists. In the year following the Crown report, group protocol arrangements were given full legal status, ensuring another formal route to attain access to medicines by AHPs [59-61].

#### 2. Australasia

Similar shifts in health policy reform occurred, in a comparable timeframe, in Australia, both at state and territory, and federal (Commonwealth) level [37,39]. Provision of adequate care for an ageing population, increased costs of medical technology (and new medications) and a crisis in recruitment and retention in healthcare services were also relevant to Australian healthcare [2,37]. Role boundary 'flexibility' and a reconfiguration in the healthcare workforce became a central facet of Australian health policy in the last decade, with high level discussions on expanding the use of role substitution [37,54,55]. Discussions to emerge from the Australian Health Minsters' Conference of 2004 laid the foundations for the National Health Workforce Strategic Framework, highlighting the immediacy of concerns surrounding health workforce shortages involving all of Australia's State, Territory and Federal Health Ministers [37]. Similar concerns were expressed in the Australia Institute of Health and Welfare reports at that time [62]. In late 2005, the Australian Productivity Commission published its research report, 'Australia's Health Workforce', which further affirmed the need for role flexibility and reform of traditional health provider roles within the Australian health system [63]. However, the recommendations in the report were largely ignored by the Howard government, and the emphasis on role substitution was condemned by some elements within the medical profession [64,65], despite significant support within the mainstream press and the other sections of the medical profession, along with consumers, nursing and allied health professions [66-68].

The granting of authority for professions to incorporate drug prescribing into scope of practice in Australia is complicated by separate and individual state and territory 'drugs and poisons' legislation. There is no overarching Commonwealth legislation to provide national governance to prescribing, so any emerging profession seeking prescribing amendments has needed to repeat this process in every state and territory. Given the peculiarities of individual state and territory legislation and policy, non-medical professions have seen inconsistent and variable formularies and governance develop across the country, in contrast to the medical profession. It is interesting to postulate that, in the light of the incidence of adverse events relating to medical prescribing now known, whether the regulators of an earlier era would have conferred such broad prescribing rights even for medicine.

However, funding for pharmaceutical provision in Australia is achieved more simply at national level, via the Pharmaceutical Benefits Scheme (PBS), and is a Commonwealth government responsibility. Unlike the UK system, there is no provision for AHP (or nurse practitioners or doctors) prescribing from a local primary health care budget. Currently, approximately 80% of prescriptions dispensed in Australia are covered by the PBS scheme, which has only as recently as 2007 included a budget for prescriptions written by optometrists (though no other non-medically qualified professions, except dentistry), and is growing at a rate of 10-15% annually [69].

Access to PBS funding for non-medical prescribing groups remains the last barrier to equitable access to prescription medications for patients of non-medically qualified professions in Australia. This fact has not been lost on the Australian Medical Association, which remains staunchly opposed to non-medical prescribing and PBS reforms, and argues that "the slippery slope to doctor pretenders is well and truly with us and although there are a variety of pretenders with a variety of agendas, the successful agenda is pretty much always the same...It is part of a much broader push towards task substitution which the AMA has under the magnifying glass..." [70]. As optometrists (and imminently nurse practitioners) have statutory rights in all states and territories to prescribe restricted drugs under the PBS, it is reasonable to suggest that only professions that have successfully lobbied to amend all individual 'drugs and poisons' legislations may be likely to receive Commonwealth support for PBS benefits.

With the emergence of the Rudd Government into federal government in 2007, the move towards a health reform agenda became a key Labour policy at a national level, although many of the policy initiatives had been instigated by the previous administration. By 2008, the Council of Australian Health Ministers had moved ahead with this reform agenda, leading to the establishment of the National Health Workforce Taskforce, and a program for National Registration and Accreditation for the majority of health professions. Combined with these activities, the Rudd Government also announced the establishment of system wide reviews of the health system, including the National Health & Hospitals Reform Commission and the National Primary Health Care Strategy, to investigate options for review of the health system.

In an unanticipated move, the Rudd Government sought to pre-empt the outcomes of these reviews in the 2009-10 federal Budget papers, by supporting registered nurse practitioners and midwives under the Medicare Benefits Schemes (MBS) and PBS Scheme, along with making provision for public indemnity insurance for midwives working within hospital settings. This move was set to directly affect medical specialists, as some existing MBS rebates (for services such as In Vitro Fertilization and ophthalmology) were proposed for reduction under these budget provisions - in order to fund Commonwealth supported nurse practitioner and midwife activities. In proposing this legislation, the Health Minister, Nicola Roxon, commented that it was "one of the centrepieces of the Rudd Government's workforce and primary health agenda" and, "a landmark change for Australia's nurses and midwives"[71]. The Australian Medical Association, however, remained opposed to the relevant Bill, proposing in a submission that it be amended to include a requirement for medical practitioners to be 'gatekeepers' to nurse practitioners and midwives (thus excluding direct access from the public), along with greater oversight and a 'sunset' clause [72].

The pattern of regulatory approval for New Zealand podiatrists to access prescription medications bears many similarities to that of the UK and Australia. In 1975 podiatrists obtained the right to administer the local anaesthetic lignocaine, and since this time has been reported by the registration authority to have been without incident or deleterious outcome for any patient [73].

In 2002, the New Zealand New Prescribers Advisory Committee was established under Section 8 of the Medicines Act 1981, to examine the role of prescribing by health professions. The role of this committee was to assess applications for extending limited independent prescribing authority to new groups of health practitioners in New Zealand and provide recommendations to the Minister of Health, until it was disbanded in 2006 and taken over by the Ministry of Health.

### Podiatry in the UK and Australasia

In both Australasia and the UK regulatory and legislative change has been gradual, but, as an AHP, podiatry is acknowledged as fully involved in the administration, access, supply, and prescription of prescription only and pharmacy medicines. Indeed, key shifts in the role and task domains within podiatry have evolved considerably over the years, and have been directly related to access to medicines.

In Britain, the immediate impact on podiatry of the Medicines Act (1968) was to undermine normative practices, such as drug preparation, and bar access to local anaesthetics [28]. The potential consequences raised considerable anxiety across the profession [34]. Prolonged and difficult lobbying, extending over a four year period, finally led to approval from the regulatory authority for the use of local 'analgesic' techniques, but still did not enable legislative access to the medicines [28]. Access was not fully obtained until 1980, following an even longer period of lobbying [74]. Protracted negotiations involving proposals and counter proposals were marked by arguments over dosages and concentrations of solutions, before agreement was finally reached [28].

Acquisition of rights to local anaesthesia opened the door to the ongoing development of podiatric surgery, which would clearly have had difficulty continuing without access rights [34]. Administration techniques were also rapidly expanded, from simple toe anaesthesia techniques, to full foot ankle block techniques, enabling more complex procedures to be undertaken. Indeed, most podiatric surgical procedures continue to employ local anaesthesia methods [75].

The Thatcher Government, and the John Major administration that followed, signalled a major shift in healthcare policy, drawing on neo-liberal principles in introducing deregulation and competitive tendering for contracts in healthcare provision - a climate that engendered the prospect of further change in the medicines legislation [47,52]. The Society of Chiropodists canvassed its membership and collated evidence on the extent and scope of medicines usage, in a bid to construct a new, evidence-based, submission. The evidence, drawn from referral patterns, pointed to a need for access rights to certain oral antibiotic agents, notably erythromycin and flucloxacillin as well as a defined and limited range of other prescription only and pharmacy medicines [76]. Even the regulatory body acknowledged the request as legitimate, based as it was on evidence drawn from the membership, noting that the referral patterns for those prescription only medicines sought (especially antibiotics) were 'regular'. An enhanced role for podiatrists in the field of medicines, as well as surgery, was further acknowledged by the Department of Health, in a joint NHS Chiropody Task Force publication of 1994 [77]. Considerable emphasis was placed on the logic used to justify the proposed extensions, focusing on easing the patient pathway and reducing GP workload by preventing duplication of effort. In doing so, the submission adhered to the principles of the wider policy agenda, promoting a smooth, collaborative, inter-professional approach to patient care [28]. In spite of the evidence and logic, the resulting exemption order reflected only limited success. Whilst access to several more prescription only and pharmacy medicines was granted, others, such as the antibiotics on the list, were denied [78,79].

The formalisation of patient group directions (which had been operating previously as group protocols) added another tier to the options available to podiatrists to access restricted category medicines. Podiatric surgeons probably benefited more than most in adopting this mechanism within the National Health Service, using it to gain access to a wide range of prescription only medicines beyond the scope of the existing exemptions, and thus further facilitating extensive foot surgical procedures. In addition, it continued to appeal to the modernisation agenda, establishing role flexibility and thus enabling greater access to medicines by patients [80]. Podiatrists specialising in diabetes care or rheumatology were also increasingly able to access these mechanisms in order to ease patient throughput in complex multi-professional hospital clinics, reducing demand on hard pressed physicians and allowing the development of new skills, such as intra-articular injections. Patient group directions, were, however, locally devised and agreed, and were thus only possible to enact with the co-operation of those physicians willing to engage with the process, leading to disparities across the country. AHP prescribing was further acknowledged by the introduction of enabling legislation in the form of the Health and Social Care Act (2001), acting as primary legislation, and thus superceding key sections of the Medicines Act (1968) [81].

Many physicians were highly supportive of expanding the role boundaries of allied health workers, including podiatrists, most notably within the diabetes fraternity. Some even suggested a role for podiatrists in the treatment and management of hypertension and insulin dose alteration [82]. However, universal support is lacking, and the use of patient group directions is widely viewed as a measure likely to be replaced by a more robust system in due course, such as independent prescribing. In some measure this more robust process has already been established as 'supplementary prescribing', extended to physiotherapists, radiographers and podiatrists in April 2005 [83]. However, it has received mixed responses from within the profession, being effective in multi-professional environments, but less effective in independent practice, especially in podiatric surgery, possibly accounting for the limited uptake [84]. Indeed, by 2008 only 64 podiatrists had become supplementary prescribers [85]. It is, in part, possible to account for this finding, as opportunities to undertake training in supplementary prescribing are 'rationed' by employers, who are required to fund places and provide mentorship.

AHP 'supplementary prescribing' also has the disadvantage that it is dependent upon co-operative physicians, who are essential as both mentors in training and as independent prescribers in practice. Without an authorised initial diagnosis and clinical management plan it is not possible to utilise a supplementary prescriber. Nevertheless, although it was originally envisaged that the supplementary prescribing role would consist of monitoring and adjusting existing prescriptions, in practice it has proved sufficiently flexible to enable the care of acute medical emergencies in patients with chronic illness (such as infected ischaemic ulcers in cases of diabetes) [85].

In 2006 a new exemption list for podiatrists was introduced reflecting further the impact of health policy modernisation and the diminishing authority of medicine over prescribing. It included full access to the antimicrobials amoxicillin, flucloxacillin and erythromycin, without any specification on dosage or route of administration [86]. It affords podiatrists in general practice the ability and scope to deploy antimicrobials to combat infections and to access adrenalin for use in emergency circumstances. Reflecting on the failure of repeated previous attempts to gain access to these medicines, the 2006 exemption particularly illustrates the new climate of change, and the growing acceptance of the reality of workforce redesign and role transfer in the sphere of medicines.

Most recently, in July 2009, the UK Department of Health published a report for the Chief Health Professions Officer, examining the case for extending prescribing and medicines supply mechanisms for the allied health professions [85]. It concluded that there was a 'strong case for progression to independent prescribing for physiotherapists and podiatrists', and included key recommendations that further work be undertaken to establish independent prescribing for these two groups. Independent prescribing for podiatrists, whilst not directly comparable to medical or dental prescribing, is, seemingly, very much on the agenda. The change of climate is consistent with the need to develop a workforce capable of taking on new, expanded roles previously within the exclusive domain of medicine [87,88]. It is nevertheless intriguing to note that there remains an important distinction between independent prescribing for the AHPs, and medical (or dental) prescribing. Unlike the latter, the former does not include access to unlicensed medicines or controlled drugs (the equivalent of S8 in Australia). At face value this may seem relatively unimportant, yet podiatrists continue to use, very widely, at least one agent that until recently was regarded by the Medicines and Healthcare Products Regulatory Agency as an unlicensed medicine - specifically liquefied phenol, used in toenail ablation techniques. In July this year the Medicines and Healthcare Products Regulatory Agency published a statement asserting that, as it "does not have a primary mode of action which is pharmacological, metabolic or immunological it falls outside the definition of a medicinal product". As a result, providing the product is not marketed with medicinal claims, it is no longer subject to medicines legislation. In combination, these recent changes reflect the pace of the broader workforce trends towards redesign, role substitution and enhanced flexibility.

Like the UK, administration rights to local anaesthetic agents (Schedule 4 drugs) became the first marker of change for podiatry in Australia, facilitating the same advances in practice and similar challenges from the medical profession [37]. Recent data shows that each State possesses similar access to a range of local anaesthesic agents, achieved over a comparable timeframe to that in the UK, where South Australia appears the most liberal, and (up until recently) Queensland the most restrictive [89]. Although the schedules vary from state to state, only in South Australia, Western Australia and, more recently, Victoria and Queensland, are access, administration, supply or prescription rights to restricted or controlled medicines available (Schedule 4 or 8 medicines), though these are largely restricted to the relatively small workforce of qualified podiatric surgeons.

It is notable that since the original legislation, only South and Western Australia have seen subsequent additions and modifications. Furthermore, the Adverse Drug Reactions Advisory Committee has indicated that there is "no known pattern of adverse reactions relating to podiatric prescribing", although the Australian Medical Association in Victoria openly disputed this, indicating that "the suggestion that there has been no adverse side effects to medications prescribed by podiatrists reflects the hubris of many non-medical professions who seek prescribing rights..." [90]. This criticism does raise the suggestion for the profession and its governing authorities to develop, or integrate into existing, adverse-event reporting pathways, and for public data to be collected on podiatric prescribing in Australia.

In Australia, access to medicines is governed by a 'drugs & poisons' authority in each State and Territory, although the actual 'scheduling' of medicines is a Commonwealth (Federal) responsibility, undertaken by the National Drugs & Poisons Schedule Committee (a branch of the Therapeutic Goods Administration and equivalent to the UK's Medicines and Healthcare Products Regulatory Agency) in combination with the Commonwealth Department of Health and Ageing [37,39]. Specific regulation on the mechanisms for the supply, administration or prescription of 'restricted and controlled' drugs is contained in a number of State & Territory 'drugs and poisons' legislation (for eg. Poisons Act 1933; Poisons and Drugs Act 1978; Drugs of Dependence Act (1989). Unlike the UK, there is no single over-arching medicines legislation, although currently plans to introduce a uniform scheduling of medicines to effect 'harmonisation' across Australia and New Zealand are underway [91]. A new Medicines and Poisons Bill (2006) is currently under consultation, and will "not change non-medical prescribing rights" but will grant "consideration...to a proposal to grant ACT podiatrists limited prescribing rights" [39].

In South Australia rights to a limited list of restricted medicines (other than local anaesthesia) were granted in 1989, and extended in 1996, largely limited to qualified podiatric surgeons. Similar changes were established in Western Australia in 1995, where podiatrists with a relevant Master's degree were able to apply to supply (but, importantly not prescribe) a narrow range of restricted drugs such as antibiotics and analgesics.

In Queensland, amendments to the Health (Drugs & Poisons) Regulation 1996 came in 2006 to allow recognised 'surgical podiatrists' (who hold Fellowship with the Australasian College of Podiatric Surgeons) to prescribe, supply or administer a limited formulary of Schedule 4 and one Schedule 8 drug. Importantly, Queensland then is the only Australian jurisdiction to allow authorised (surgical) podiatrists to prescribe a 'controlled' S8 drug of dependence (oxycodone), for managing postoperative pain. Additionally, the amendments allowed for general podiatrists to access adrenaline (in a pre-loaded device) for the emergency management of anaphylaxis, though curiously not for use in combination with local anaesthesia (as it is in several other Australian jurisdictions), unless the registrant was an endorsed 'surgical podiatrist'. Additional plain preparations of several other local anaesthetic agents were also made available for administration by general podiatry registrants.

It is in Victoria that the most recent, and advanced rights have been attained. Under the terms of the Health Professions Registration Act (2005) and the 2007 Regulation amendments to the Drugs, Poisons and Controlled Substances Act 1981, the Podiatrists' Registration Board had been given authority to determine which Schedule 2,3 and 4 medicines may be possessed, used, sold or supplied by its registrants following approval by the Health Minister. As a result, the Podiatrists' Board was empowered to create a subset of registrants known as 'authorised prescribers'. The Acts do not specify the particular form of undergraduate or postgraduate training, leaving these decisions to the Podiatrists Board, via advice from its own Prescribing Practice Advisory Committee and key stakeholders, and in consultation with the Minister. Most significantly, the Schedule 2, 3 and 4 drugs approved in Victoria are available for use by all suitably qualified podiatrists, and not just podiatric surgeons, as is predominantly the case elsewhere. Final approval of the initial formulary was given by the Health Minister in June 2009.

Under the current process of *National Registration & Accreditation*, local state and territory health professional registration boards will be disbanded and replaced by national authorities. As such, the Podiatry Board of Australia was constituted in 2009, with the task of taking over the administration of registration and regulation of standards of practice for all Australian podiatrists in July 2010. Under the requirements of the *Health Practitioner Regulation (Administrative Arrangements) National Law Act 2008*, the Board has already begun consultation on the mechanisms for the arguably overdue implementation of national standards for podiatric prescribing within Australia, to be submitted for approval by the Australian Health Workforce Ministerial Council [92]. However, the move to any national prescribing standard will still be adversely affected by the jurisdiction inconsistencies of local drugs and poisons legislation in different states and territories, and a uniform approach will be a highly desirable long term solution to addressing this problem.

In 2005 a joint application was made by the Podiatrists Board of New Zealand and the New Zealand Society of Podiatrists to the New Prescribers Advisory Committee for podiatrists to be recognised as "designated prescribers," in the Regulations under the Medicines Act 1981[73]. As part of this application, the proposed curriculum for New Zealand registrants wishing to potentially become a 'designated prescriber' was put forward from the Auckland University of Technology in the form of a Postgraduate Diploma of Health Science modelled on the nurse practitioner curriculum.

Under the terms of the Health Practitioners Competence Assurance Act 2003, the Podiatrists Board of New Zealand was granted the authority to determine Scopes of Practice for the profession. In doing so, it determined a new category of advanced scope practitioner known as a 'podiatric prescriber' [73]. However, as at the end of 2007, the Podiatrists Board of New Zealand announced that NPAC had accepted its submission in principle, pending final modifications to the proposed monitoring processes and final list of medications, prior to activating the 'podiatric prescriber' category of registration [93]. Table 1 summarises the varying and inconsistent nature of the various requirements for endorsed podiatric prescribers in Australasia.

**Table 1:** Summary of statutory requirements for drug prescribing by podiatrists in Australasia (as at 2009)

Jurisdiction	Educational requirements for prescribing	Governance level
**Victoria**	Recent undergraduate podiatry degree (2003 onwards from Latrobe University), with Board approved postgraduate pharmacology studies and clinical experience [interstate or less recent graduates are required to undertake additional core content studies and clinical experience]	*Least restrictive*

**New Zealand**	A postgraduate qualification as determined by the Podiatrists Registration Board of New Zealand, or equivalent overseas qualification	

**Western Australia**	Master's degree with advanced pharmacology core unit	

**South Australia**	Fellowship of the Australasian College of Podiatric Surgeons	

**Queensland**	Fellowship of the Australasian College of Podiatric Surgeons + additional Board requirements	*Most restrictive*

## Discussion

It is clear that the recent changes in prescribing rights for AHPs in both the UK and Australasia reflect the impact the forces of neo-liberalism, new public management and economic rationalism have had on medical autonomy in the arena of prescribing. The trend towards workforce flexibility and role substitution has led to enhanced roles for the AHPs, and this has been extended to the prescribing arena, which, of course, is one of the most distinctive task jurisdictions that medicine has traditionally controlled. By examining the case exemplar of podiatry, it has been possible to trace the earlier attempts, from the 1960s and 1970s, of this group to secure a foothold in the area of medicines, and to contrast this early paucity of success with later developments. Indeed, the rapidity of change in the last decade bears no resemblance to the tortuous and near futile efforts of a decade earlier to achieve meaningful prescribing rights.

Yet, opposition by the medical profession has been fairly consistent in both the UK and Australasia. Whilst this opposition may have been moderated in the UK, in the light of health policy reform, it is much less obviously so in Australia [94]. Although, as recent changes enabling the independent prescribing status of some nurses and pharmacists suggests, non-medical prescribing may be an integral and irreversible part of the changing landscape of modern professionalism, it is also premature to suggest that the authority of medicine in influencing and determining the content of work of other health professions is at an end. New modes of prescribing available to the majority of AHPs remain in several ways subject to the authority of medicine (such as patient group directions or supplementary prescribing, which require the written authority of the doctor, or the doctor's mentorship, or delegation from the doctor once the diagnosis and management plan has been decided).

In both the UK and Australasia, allied health professionals access, administration and prescribing rights have been subject to limitation - either in the ways described above, or simply in the limited lists or formularies that require extensive effort and legislative approval to modify. Only in independent forms of prescribing is clinical or technical autonomy fully exercised in the prescribing field, although amendments and statutory instruments altering specific professions access and administration rights do, in effect, confer some degree of autonomy - yet these are difficult to obtain and usually require lengthy periods of lobbying in advance. Also significant in the broader picture is the extent to which educational advances within the profession have enabled further rights and a greater scope of prescribing practice, acknowledged by regulators in both the UK and Australasia. In Australia, there is little doubt that the additional and extensive training required to practice as a podiatric surgeon underpinned wider access to restricted medicines since the 1980s.

## Conclusion

In constructing a chronological account of 'prescribing' within the profession of podiatry in both Australasia and the UK, grounded in a socio-historical context, it has been possible to demonstrate the influence of health policy drivers at work in determining change, and to highlight, therefore, the rapidity and extent of the changes within the last decade. The reality of workforce redesign is amply illustrated in the case of AHP prescribing, and constitutes one facet of the broad policy agenda intended to ensure a new health service provision, fit for purpose in the 21^st ^century. Clearly, the AHPs must rise to the challenge.

## Competing interests

The authors AS, SAN and RB declare that they have no competing interests. AMB is currently Deputy Editor-in-Chief (UK) of *Journal of Foot and Ankle Research*. It is journal policy that editors are removed from the peer review and editorial decision making processes for papers they have co-authored.

## Authors' contributions

All the authors were involved in the conception and design of the work within the paper. AMB provided the main UK perspective, AS, SAN and RB data on the Australian perspective. AMB and AS initially drafted the manuscript, with critical revision and essential ongoing advice from SAN and RB. All authors contributed to the interpretation offered.
